# Improving the use of crop models for risk assessment and climate change adaptation

**DOI:** 10.1016/j.agsy.2017.07.010

**Published:** 2018-01

**Authors:** Andrew J. Challinor, Christoph Müller, Senthold Asseng, Chetan Deva, Kathryn Jane Nicklin, Daniel Wallach, Eline Vanuytrecht, Stephen Whitfield, Julian Ramirez-Villegas, Ann-Kristin Koehler

**Affiliations:** a Institute for Climate and Atmospheric Science, School of Earth and Environment, University of Leeds, Leeds LS2 9JT, UK; b CGIAR-ESSP Program on Climate Change, Agriculture and Food Security, International Centre for Tropical Agriculture (CIAT), AA 6713 Cali, Colombia; c Potsdam Institute for Climate Impact Research, 14473 Potsdam, Germany; d Agricultural & Biological Engineering Department, University of Florida, Gainesville, FL 32611, USA; e Institut National de la Recherche Agronomique (INRA), UMR AGIR, BP 52627, 31326 Castanet Tolosan Cedex, France; f Division of Soil and Water Management, Department of Earth and Environmental Sciences, KU Leuven, Celestijnenlaan 200E, P.O. 2411, B-3001 Heverlee, Belgium

**Keywords:** Crop model, Risk assessment, Climate change impacts, Adaptation, Climate models, Uncertainty

## Abstract

Crop models are used for an increasingly broad range of applications, with a commensurate proliferation of methods. Careful framing of research questions and development of targeted and appropriate methods are therefore increasingly important. In conjunction with the other authors in this special issue, we have developed a set of criteria for use of crop models in assessments of impacts, adaptation and risk. Our analysis drew on the other papers in this special issue, and on our experience in the UK Climate Change Risk Assessment 2017 and the MACSUR, AgMIP and ISIMIP projects.

The criteria were used to assess how improvements could be made to the framing of climate change risks, and to outline the good practice and new developments that are needed to improve risk assessment. Key areas of good practice include: i. the development, running and documentation of crop models, with attention given to issues of spatial scale and complexity; ii. the methods used to form crop-climate ensembles, which can be based on model skill and/or spread; iii. the methods used to assess adaptation, which need broadening to account for technological development and to reflect the full range options available.

The analysis highlights the limitations of focussing only on projections of future impacts and adaptation options using pre-determined time slices. Whilst this long-standing approach may remain an essential component of risk assessments, we identify three further key components:

1. Working with stakeholders to identify the timing of risks. What are the key vulnerabilities of food systems and what does crop-climate modelling tell us about when those systems are at risk?
2. Use of multiple methods that critically assess the use of climate model output and avoid any presumption that analyses should begin and end with gridded output.
3. Increasing transparency and inter-comparability in risk assessments. Whilst studies frequently produce ranges that quantify uncertainty, the assumptions underlying these ranges are not always clear. We suggest that the contingency of results upon assumptions is made explicit via a common uncertainty reporting format; and/or that studies are assessed against a set of criteria, such as those presented in this paper.

## Highlights

• 14 criteria for use of crop models in assessments of impacts, adaptation and risk
• Working with stakeholders to identify timing of risks is key to risk assessments.
• Multiple methods needed to critically assess the use of climate model output
• Increasing transparency and inter-comparability needed in risk assessments

## 1 The role of crop models in assessing risk and adaptation

Crop models have a long history, during which their focus and application have altered in response to societal needs (Jones et al., 2016). They have contributed to decision support (e.g. Kadiyala et al., 2015) and risk assessment (e.g. Rader et al., 2009), and have resulted in conceptual and practical advances in publicly-funded agricultural development work (Reynolds et al., this issue). The last decade has seen an increase in the use of crop-climate ensembles targeted at informing adaptation (e.g. Challinor et al., 2013). Much of the progress made has been enabled by model intercomparison projects (MIPs). The Agricultural Model Intercomparison and Improvement Project AgMIP (Rosenzweig et al., 2013b), the Inter-Sectoral Impact Model Intercomparison Project ISIMIP (Warszawski et al., 2014), and Modelling European Agriculture with climate change for Food Security MACSUR (Bindi et al., 2015) have brought together large model ensembles that are run for different sites and crops or in gridded form for larger areas or globally.

Food systems risks can be defined narrowly as the potential for reduced food production (e.g. Li et al., 2009), or broadly as the risk to food security. Even more broadly, food systems have many interactions with other systems, e.g. the energy system (Homer-Dixon et al., 2015). Crop models will have a greater or lesser role in the analysis, depending on the nature of the risks being assessed. The UK Climate Change Risk Assessment (CCRA2017)^1^ aimed to identify all the climate risks requiring action by the UK government – i.e. all those that are not addressed by current policy. Topics covered in CCRA2017 include domestic food production and international dimensions of risk, including food security, conflict, migration and humanitarian aid, and their inter-relationships (see Challinor et al., 2016b).

Integrated assessment of risks from climate change is a relatively recent focus for crop modelling. Ewert et al. (2015) have set out a valuable review and outlook for risk assessment using crop models as part of integrated assessment models. Here, we examine the use of crop models for risk assessment outside of this emerging field. We draw on author experience in both MIPs and CCRA2017. Our analysis is also based on a list of criteria for application of crop modelling to impacts, adaptation and risk assessment; and on a list of identified research priorities for the crop-climate modelling research community. These lists, which can be found in Section 1 of the supplementary information, were developed first amongst the authors and then distributed more widely amongst all authors of this special issue, to ensure feedback and consensus. The manuscript reviews were also used to refine the lists.

**Fig. 1:**
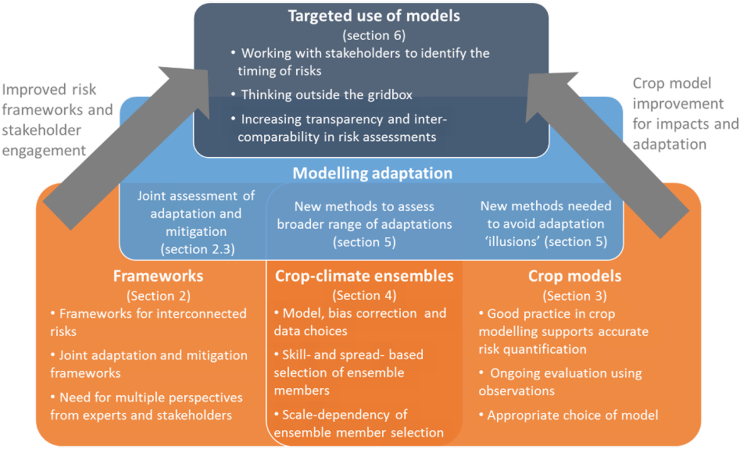
Summary of key issues identified by our analysis. The structure shows how fundamental work on frameworks, crop models and ensembles are used to improve adaptation studies and ultimately target models towards stakeholder-relevant risk assessments.

Our analysis reviews and assesses the frameworks needed for risk assessment (Section 2); the development and running of crop models (Section 3); the methods used to form crop-climate ensembles (Section 4); and the methods used to assess adaptation (Section 5). Good practice in all of these areas underpins accurate risk assessment. We conclude with a forward-looking assessment of how crop models might be better used to improve risk assessments (Section 6). The key issues identified in our analysis are presented in Fig. 1.

## 2 Towards improved framing of risks posed by climate change to food production systems

### 2.1 Risk, uncertainty and likelihood

Risk and uncertainty are concepts that apply where the range of future possibilities is largely known (Stirling, 2010). The difference between them lies in whether or not probabilities can be calculated (Wynne, 1992). This distinction is often a matter of (expert) opinion rather than provable fact, so that the same crop-climate ensemble can be presented as an assessment of risk or as an assessment of impacts expressed using uncertainty ranges. True assessment of risk implies a knowledge of the consequences of an event, since risk is the product of two factors: the probability that an adverse event will occur and the consequences of that adverse event (Jones, 2001). For simplicity, however, and following the conventional use of the term “risk” in much of the crop modelling literature, we do not distinguish here between likelihood and risk. Clearly any contribution to the assessing likelihood can be a component of a risk assessment.

### 2.2 Frameworks for interconnected risks

Interactions between sectors (e.g. agriculture, forestry, water) are important in determining climate change impacts (Harrison et al., 2016, Elliott et al., 2014, Piontek et al., 2014). In CCRA2017, a very broad systems boundary was needed in order to draw the most robust conclusions possible. Where quantitative information on interactions was not available, those relationships were assessed using existing literature. Studies that focus on interactions often fill key knowledge gaps. Guzman et al. (this issue) provide an exemplar study of interactions between crop cultivation, irrigation and groundwater. Elliott et al. (this issue) provide an exemplar study of economic impacts by assessing the insured and uninsured crop losses resulting from drought.

**Fig. 2:**
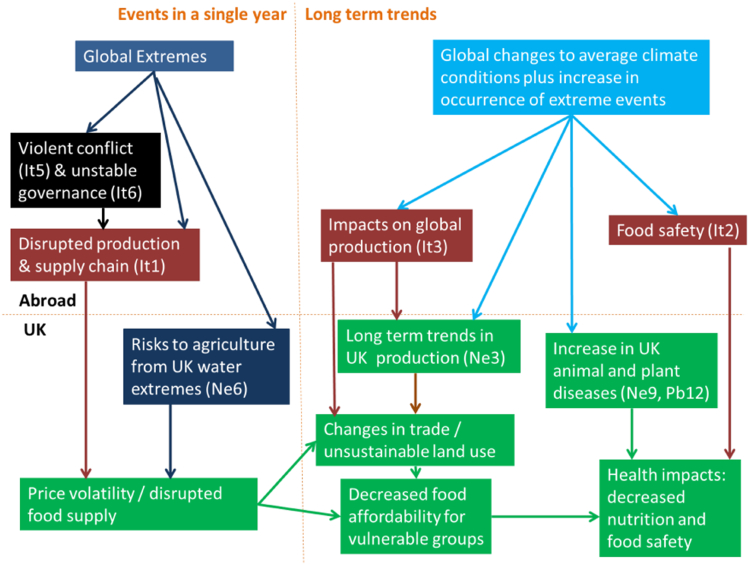
Risks to UK food systems derived from an analysis of international (“It”) and domestic dimensions of climate change. Domestic dimensions arise from risks to natural environment and natural assets (“Ne”) and people and the built environment (“Pb”). Blue indicates climate change; green shows impacts on UK food systems and society; brown shows international food system risks that are transmitted to the UK; black indicates factors that compound international food system risks. Full details, together with the other enumerated lists, are contained in Challinor et al. (2016), Brown et al. (2016) and Kovats et al. (2016), and via interactive web resources at UK Committee on Climate Change (2016). (For interpretation of the references to color in this figure legend, the reader is referred to the web version of this article.)

The interactions that lead to climate change risks go beyond those amongst ecosystem-based sectors and into governance, society, health and economics, to name but a few areas. Fig. 2 summarises those findings of CCRA2017 that relate to food security (Challinor et al., 2016b). Key issues that emerged in that assessment are the fundamental interconnectedness of both climatic and non-climatic risks and the transmission of risks across international boundaries (e.g. transnational transmission of risks to crops from ozone Hollaway et al., 2011). Thus, the relevance of crop modelling goes well beyond an understanding of food production, or even food security, and there is a concomitant breadth required in the systems boundaries used in crop modelling studies (Campbell et al., 2016, Waha et al., 2012), especially where broad system boundaries are used.

Integrated assessment models (IAMs) may be expected to deliver frameworks for interconnected risks; however the use of crop models within IAMs is at a relatively early stage (Ewert et al., 2015). Further, IAMs may not be the best tool to assess the range of trade-offs and synergies that are important to food systems. The complexity of the inter-related set of climate change and food security risks and responses has led to them being labelled a “wicked problem” requiring a range of approaches (Vermeulen et al., 2013). Food security targets are not solely a matter of increasing yield, but also of improving food access, quality and diversity. There may be direct yield trade-offs involved in actions and activities that contribute towards food security (Campbell et al., 2016). The integration of local knowledge and the input of social scientists within interdisciplinary modelling research can contribute to the identification and outlining of realistic scenarios of socio-technical change, crop-climate indices, or of model output priorities (i.e. not solely yield Herrero et al., 2015, Campbell et al., 2016). The insights gained may inform the design of models and modelling studies that go beyond conventional projections of yield and yield response and are designed to analyse trade-offs (Wessolek and Asseng, 2006), determine least regrets options, or inform multi-criteria analyses (Hallegatte, 2009, Challinor et al., 2010).

### 2.3 Joint adaptation and mitigation frameworks

Much of the current focus on assessing the risks of climate change is focused on the stringent 1.5–2 °C limit on global warming agreed at the international climate negotiations in Paris in 2015 (COP21). In order to be consistent with a 2 °C target, emissions across all sectors need to decrease by over 80% by 2050 (Edenhofer et al., 2012), with even greater reductions required for a 1.5 °C target. The agriculture, forestry and other land use sector is responsible for 24% of all human greenhouse gas (GHG) emissions (Smith et al., 2014), so is a critical sector for delivering the Paris Agreement. More than even before, it is clear that agricultural systems require changes that address both adaptation and mitigation.

Both sustainable intensification and climate-smart agriculture (Lipper et al., 2014) seek to address the challenge of joint adaptation and mitigation challenge. Climate-smart agriculture targets the simultaneous achievement of increasing agricultural production, adapting to climatic change, and mitigating this change through reduced agriculture-related emissions. Understanding and addressing the trade-offs and synergies between these objectives is therefore a research priority for the climate-crop modelling community (Campbell et al., 2016), which is particularly well placed to contribute given its capabilities to simulate regional and global scale change.

How might the crop-climate modelling community develop joint adaptation and mitigation frameworks? One approach would be to calculate, or at least estimate, the emissions associated with modelled adaptation options. Tian et al. (this issue) exemplify this approach by quantifying the non-CO_2_ greenhouse gas emissions associated with different paddy rice management strategies and examining yield-emissions trade-offs. Composite measures, such as yield emission efficiency, might also be used to assess how climate-smart specific adaptation options are. A set of recent studies exemplify different existing frameworks for the joint assessment of adaptation, productivity and mitigation outcomes for different types of agricultural interventions, technologies and practices (e.g. Shirsath et al., 2017; Shikuku et al., 2017; Notenbaert et al., 2017).

### 2.4 Risk frameworks need to incorporate multiple perspectives

In addition to being a technically challenging issue, understanding risk and uncertainty requires cognisance of the multiple perspectives and interpretations that exist (Wesselink et al., 2014). The frameworks used to conceptualise uncertainty determine the potential for crop-climate modelling to distinguish risks. A range of interpretations on these related topics exists not just between different groups (scientists, politicians, public), but also within them. Even experts within the same project can disagree on the meaning and adequacy of reported uncertainty ranges, based on their assessment of whether or not all risks are known and whether or not the known risks are adequately quantified (Wesselink et al., 2014; see also Section 2.1).

**Fig. 3:**
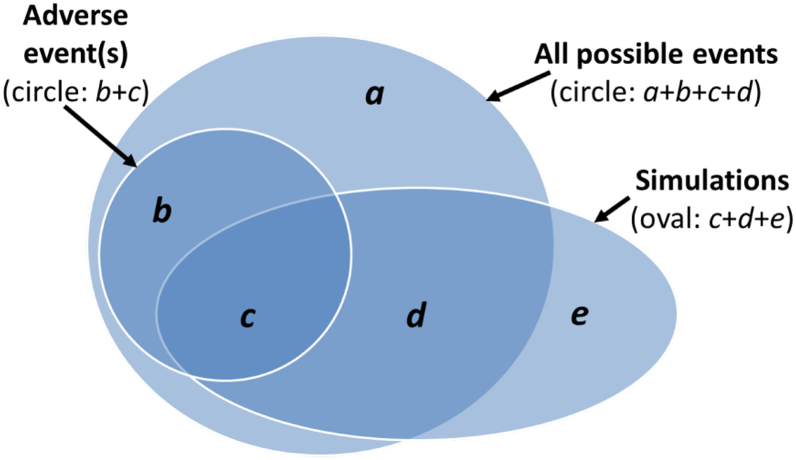
Schematic representation of risk assessment. The large circle (a + b + c + d) shows all the possible realisations of a variable of interest (e.g. crop yield, food prices, mycotoxin contamination). Of this full set a smaller circle (b + c) shows those realisations that are judged to present a risk. Of these adverse events, only area (c) is captured by the simulations. Of the remainder of the possibilities, only area (d) is captured by the simulations. Additionally, area (e) shows the set of unrealistic simulations.

Systematic assessment might seem to be a way to ensure objectivity. However, herein lies the thorny issue at the heart of uncertainty analysis: attempts to be systematic, for example by quantifying parametric uncertainty by using ranges of values, can result in ranges that are not informative, and even unrealistic (Challinor et al., 2007). The same may be said of using multiple models. Fig. 3 illustrates this issue. The range of all simulated events is an attempt to capture all possible events, yet the overlap is not only partial; models and model ensembles are collections of methodological choices and assumptions that may not explore the full range of possibilities (Whitfield, 2013). Equally, the range of model results may extend beyond the realms of possibility (Spiegelhalter and Riesch, 2011). Hence risk assessment with models should not be reduced to the process of equating multiple model outputs with a probability distribution. If a meaningful risk is to be calculated then a framework is needed, even if it exists only to highlight the limitations of the analysis. In Fig. 3, the real risk is represented by the ratio of “Adverse” to “All possible” events (i.e. (*b* + *c*)/(*a* + *b* + *c* + *d*)), which in this case is under-estimated by the model as being *c*/(*c* + *d* + *e*).

Both simulated and actual risk are the result of uncertainty, which is the lack of precision in knowing where in the “All possible” circle the final single real world realisation will lie. The “Simulated events” oval presents the model's attempt to capture exactly the underlying distribution of probabilities – i.e. the “All possible” area, usually through simulating multiple climate and socio-economic scenarios, bias correction of climate model output, crop model calibration, model ensembles and expert interpretation.

Some clear recommendations emerge from this analysis. Since expert judgment is involved in assessing the extent to which uncertainty estimates are realistic, reporting multiple, rather than single, assessments of the confidence placed by experts in particular predictions will avoid false confidence in the results (Wesselink et al., 2014). Furthermore, descriptive (as opposed to quantitative) presentation of results, especially when based on a consensus view, can help reframe uncertainty into statements about possible risks. In crop-climate modelling, statements that describe processes and trade-offs are likely to be more informative than broad uncertainty ranges (Challinor et al., 2013).

## 3 Developing and running crop models

### 3.1 Good practice in crop modelling underpins accurate risk quantification

The results of using a risk framework will only be as good as the models and methods used within that framework. A model needs to be skilful if its assessment of risk is to be correct.

We turn now to the technical challenges of running crop models. For a long time, it has been recognised that studies using crop models need to satisfy certain criteria in order to contribute to the literature in a valuable way (e.g. Sinclair and Seligman, 2000). A more recent review found significant issues with the way that crop models are described and used for assessing climate change impacts (White et al., 2011).

Here we present an abbreviated summary of good practice in the running of individual crop models. Each of the areas is described in more detail in the supplementary information, which describes good practice in selection of input data, model calibration and documentation of models and simulations (Section 3); and model evaluation and interpretation (Section 4). The supplementary information also presents the full list of our criteria for application of crop modelling to impacts, adaptation and risk assessment.

i. The crop model used, and the processes simulated, should be of appropriate complexity given the evidence from available data and the spatial scale of the simulations (Section 4.1). This helps to avoid overtuning during the calibration process, especially if a broad array of observed data are used (e.g. yield, LAI) across a broad range of observed values. Different models were developed to address different questions. High complexity is warranted where yield-determining processes are demonstrably complex. Field scale models are often used at spatial scales greater than those at which they were developed for, implying challenges to aggregation and parameterization (see Section 4.3).
ii. The model(s) used should be evaluated using historical observed data. A broad range of data (not just yields) over a broad range of environments should be sought and used in evaluating crop models, and error checking of the data is important. Attention to interannual variability is particularly important (see e.g. Hoffmann et al., this issue, and Müller et al. (2017)).
iii. The simulations carried out should be documented in sufficient detail to demonstrate the extent of good practice, and to ensure reproducibility of the work carried out.

### 3.2 Crop model improvement supports accurate risk quantification

With improved measurements and availability of reference data, crop models are continually being improved by more faithfully representing the processes they simulate and by identifying new processes and interactions. As long as this process does not result in unwarranted complexity (Section 3.1), this often improves skill (Maiorano et al., 2017).

Several researchers have made a case for seeking consensus amongst models and for the inclusion of N dynamics responses to elevated CO_2_ (Bannayan et al., 2005, Boote et al., 2013, Yin, 2013, Li et al., 2014). Few models (e.g. Reyenga et al., 1999, Børgesen and Olesen, 2011, Asseng et al., 2014) capture this response, yet it remains key for realistic simulation of source-sink relationships, yield quality (through protein content), sink-strength related photosynthetic acclimation to elevated CO_2_, fertilizer use, and greenhouse gas emissions from agricultural practices (Muller et al., 2014, Vanuytrecht et al., 2011).

Particularly sensitive and/or high frequency processes are another area needing improvement, since they can be especially difficult to simulate. Sensitivity studies from the AgMIP-wheat and AgMIP-rice pilot showed that uncertainty in simulated yield increased with increasing temperatures (Li et al., 2015, Asseng et al., 2013, Asseng et al., 2014). For both crops the large spread between models could be partly attributed to how phenology was simulated, i.e. the choice of cardinal temperatures, the choice of thermal time accumulation function and, for wheat, the inclusion of accelerated leaf senescence with high temperatures (Asseng et al., 2011). Similar results have been shown for potato (Fleisher et al., 2016) and for maize (Wang et al., 2015), even though this was not a general finding of the AgMIP-maize model intercomparison (Bassu et al., 2014). Furthermore, the increased uncertainty between models was due to how models dealt with an increased frequency of high-temperature events around and after anthesis and its simulated impact on crop growth.

A third area for crop model improvement is the potential need to account for microclimate, which requires simulations of canopy temperature. Recent studies have demonstrated the importance of microclimate when predicting heat sterility in rice (Julia and Dingkuhn, 2013). For wheat, canopy microclimate studies indicate that temperatures can be several degrees warmer or cooler depending on whether evaporative cooling is present (Kumar and Tripathi, 1991; Asseng et al., 2011). However, recognition of importance does not necessarily transfer into increased model skill. A study comparing nine wheat models that use three different approaches to simulate canopy temperature found only minor improvements when simulated canopy temperature was used for heat stress effects and no improvements when canopy temperature was additionally used for various other processes (Webber et al., 2017).

## 4 Crop-climate ensembles

As shown in Fig. 1, crop-climate ensembles involve both a prior modelling framework (Section 2) and the running of crop models (introduced in Section 3). We focus here on the overarching issues and approaches associated with forming a crop-climate ensemble, including choice of crop and climate models (Section 4.1), decisions on when and how to assess skill (Section 4.2), and dealing with any scale-dependency of model choice and ensemble member selection (Section 4.3). The supplementary information presents more detail on these issues, as well as the full list of our criteria for application of crop modelling to impacts, adaptation and risk assessment.

### 4.1 Forming a crop-climate ensemble

#### 4.1.1 Model and bias correction choices

The first task in implementing a risk assessment framework is to choose crop and climate models to work with. Climate model ensembles are usually chosen by the impacts community based on availability and so are to a large extent ensemble of opportunity. Similarly, crop modelling groups may have in-house crop models that they favour, often for good reasons such as confidence in their sound use of the model. However, explicit justification of model choice is often missing: White et al. (2011) found that only 18% of 221 studies reviewed thoroughly justified their choice of crop model.

Justification for use of a particular crop model in an ensemble can come entirely from a-priori reasoning – i.e. demonstration that the model is fit for purpose, as outlined in Section 3. However, in the context of an ensemble a second criterion presents itself: to what extent will that model contribute to the correct capturing of the underlying distribution of probabilities (as illustrated in Fig. 3 and outlined in Section 2)? Whilst all ensembles are to some extent ensembles of opportunity, they should not be entirely ad hoc. Rather, ensembles should be formed from a well-justified set of models and input data (see Section 2.2 of the supplementary information). As part of this, usually any climate model data used as input will be bias-corrected (see Section 2.3 of the supplementary information).

#### 4.1.2 Use of ensemble mean and spread

Ensemble mean or medians can serve as a best-estimate for the impact of climate change. Recent MIPs in crop modelling also find that the median compares better to reference data than most or even any individual model (Asseng et al., 2013, Fleischer et al., 2016, Martre et al., 2015, Bassu et al., 2014, Li et al., 2014). This result is in line with what the climate modelling community found in their model intercomparison work, which showed that the superior performance of model ensembles is a result not only of error compensation, but also greater consistency (Hagedorn et al., 2005) and robustness (Knutti and Sedlacek, 2013).

More challenging than the use of ensemble averages is the use of ensemble spread. Especially challenging, and important for risk assessments, are extreme events. Long time series of simulations are needed in order to obtain any statistical significance. Gobin et al. (this issue) address this problem by using crop models with statistical distribution fitting of extremes to assess the impact of multiple adverse weather conditions.

### 4.2 Skill-based and spread-based selection of ensemble members

Two categories of selection criteria for ensemble members can be identified: i. skill-based approaches, whereby appropriate model(s) are chosen for a targeted study, and ii: spread-based approaches, which focus on capturing the underlying distribution of possible futures using ensembles. McSweeney and Jones (2016) offer the fraction of the full range of future projections captured by a subset as a useful spread-based climate model selection metric. Ruiz- Ramos et al. (this issue), take a spread-based approach and use *ex post* expert judgment to exclude crop-climate ensemble members in a study of wheat. In contrast, Ruane et al., (2015) took a skill-based approach by justifying the selection of climate models using four criteria: spatial resolution, degree of evaluation, length of CMIP involvement, and skill in simulating monsoons.

Skill-based approaches use model evaluation statistics, whilst spread-based approaches focus on the assessment and use of ensemble ranges. As illustrated in Fig. 3, purely spread-based approaches may generate unrealistic simulations, simply because of the lack of focus on underlying model skill and evaluation. Purely skill-based approaches, on the other hand, may tend to underestimate the full range of future realisations.

Although looking at cryospheric rather than agricultural climate impacts, Wiltshire (2014) offer an interesting combination of the skill- and spread- based approaches by choosing models which are shown to best represent key features of the Indian Summer Monsoon and sample either end of the spread of precipitation projections. A more complex combination of the two approaches is recommended in Lutz et al. (2016): model selection follows a three step protocol: first, splitting the envelope of projections into four portions based upon a combination of temperature and rainfall and selecting one model from each portion (for example one model from the cold and dry portion); second, sampling of extremes; and finally filtering the remaining models based on skill in representing the annual cycle of temperature and precipitation.

Work across crop and climate modelling community can lead to improved treatments of uncertainty (Wesselink et al., 2014, EQUIP, 2014, Challinor et al., 2013). Despite the progress made with existing methods, new methods are needed for objectively determining the criteria for inclusion of models within a given multi-model study. Wallach et al. (2016) provide a valuable discussion of model selection approaches and identify a broad range of lessons for crop modellers based on methods in ensemble climate modelling. Objective criteria for model selection and weighting of ensemble members are amongst the suggestions made in that paper for improving ensemble crop modelling.

### 4.3 Scale-dependency of model choice and ensemble member selection

Choice of parameterisations (and by extension, models) that are appropriate for the spatial scale of a study is critical (see Section 2.1 of the supplementary information), since measured and modelled responses to the atmosphere can differ across scale (Challinor and Wheeler, 2008). However, in more than half of studies, models are applied at scales other than those for which they were originally designed (Ramirez-Villegas et al., 2015) – specifically, field-scale models are used above field scale in roughly 50% of the cases. Hoffmann et al. (2015), Hoffmann et al. (2016) and Zhao et al. (2015) studied the effect of using aggregated, low-resolution climate or soil input in field-scale models applied at regional scales. The extent to which model output is biased by aggregation depends upon the crop model, environmental conditions and spatial variability of weather and soil (Hoffmann et al., 2016).

Skill-based crop model selection is likely to be particularly important and possibly much easier at smaller spatial scales, where the specifics of the agroecological system being studied become increasingly important (Challinor et al., 2014a). Models often perform better in some regions than in others. This may be simply because of variation in the strength of relationships between yield and climate (see e.g. Watson et al., 2014, Watson and Challinor, 2013). However, model structure and complexity, and data and calibration issues, are also likely to play a role. The precise cause of variation in skill is difficult, if not impossible, to determine. At larger spatial scales, it is often more difficult to assess model skill, owing to scarce and uncertain reference data and aggregation issues (Porwollik et al., 2017, Müller et al., 2017). Here, spread-based crop model selection is likely to be more common.

## 5 Modelling adaptation

### 5.1 Limitations of current methods

Risk assessments will not be accurate unless they account for the autonomous adaptation that occurs in changing climates. A significant portion of the crop modelling literature has focused on assessing adaptation options: out of 91 published studies on climate change impacts used for the IPCC AR5 (Challinor et al., 2014b, Porter et al., 2014) about a third (33) also quantified adaptation. However, only four adaptation strategies were used in those studies, namely, changes in planting date, irrigation, crop cultivar and fertilizer. Adaptation studies therefore fail to represent the broad scope that adaptation has in the real world. Closing this gap is a significant challenge that very likely needs to involve stakeholders (Section 6).

Notably, little attention has been paid to changes in farm composition, including crop diversification and intercropping, which are typical of smallholding systems across the tropics (Claessens et al., 2012), as well as to long-term transformations (Rippke et al., 2016, Weindl et al., 2015). Modelled adaptations also ignore interactions within the system, e.g. changes in soil organic matter contents in mixed crop-livestock systems (Thornton and Herrero, 2015). Modelling studies also fail to represent farmers as agents who are continuously making decisions about the objectives or management of the system in the context of interacting biophysical and socio-economic drivers (Quinn et al., 2011, Below et al., 2012). As a result, framing of adaptation has skewed evidence towards a few practices and systems that can be simulated with confidence, rather than covering what is relevant in specific socio-economic or environmental contexts.

Even if the full range of adaptation options could be modelled, significant problems in quantifying adaptation benefits remain. It has been hypothesised that relative yield changes provide essentially unbiased estimates of future climate impacts that can then be applied to any technological pathway (Nelson et al., 2014, Valin et al., 2014, Springmann et al., 2016). However, any changes to agronomic management that neglect the evolution of a system under a given socio-economic pathway are unlikely to reflect the true response of the system, since they will neglect the interactions between adaptation and technological change. Similarly, crop production systems that will evolve due to technological progress and altered resource access will likely respond differently to climate change than the current systems that are typically represented in the models (Glotter and Elliott 2016). Improvement is therefore needed in the way adaptation is calculated and in the assumptions on future technologies, e.g. by employing scenarios.

**Fig. 4:**
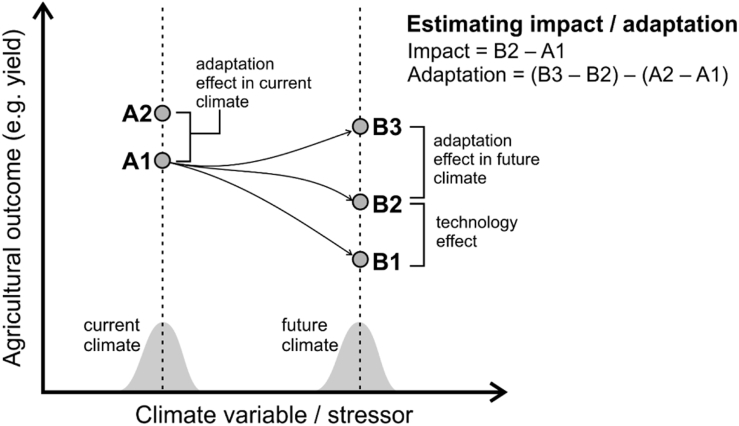
Diagram showing how crop-climate modelling studies should calculate both impacts and adaptation. A1 and A2 represent a farming system under current climate with and without adaptation (respectively), whereas B1, B2, and B3 represent the farming system of A1 but under future climate with neither adaptation nor technological progress accounted for (B1), only technological progress accounted for (B2), and with both adaptation and technological progress accounted for (B3). Based on Lobell (2014).

Modelling studies tend to compare a future with adaptation against a historical baseline, instead of comparing a climate change development pathway with its corresponding non-climate change counterfactual (Lobell, 2014). This leads to a systematic under-estimation of future crop yields. Thus, crop modelling studies typically, but not always (e.g. Ewert et al., 2005), fail to account for the technological development (often agricultural intensification) that occurs regardless of adaptation (Liu et al., 2013, Garnett et al., 2013, Tittonell and Giller, 2013). The resulting underestimation of yield is illustrated in Fig. 4 as the difference between points B1 (the future state of the system under new climate conditions, without adaptation or technological progress) and B2 (the future state of the system with technological progress, but without adaptation).

A second point for improvement regarding how adaptation benefits are quantified relates to the comparative advantage of an adaptation option under a future climate with respect to the implementation of the same option under the current climate conditions. Lobell (2014) argues that by comparing a non-adapted historical period with an adapted future period (i.e. the difference between point B3 and B2 in Fig. 4), studies tend to wrongly label certain options as adaptation, and to over-estimate the net benefit of adaptation. Future adaptation should be the result of computing the total advantage of adaptation after factoring its impact on the historical period, and its interactions with prescribed or expected technological change. Towards that aim, the crop modelling community should take advantage of recent efforts to develop scenarios of projected technological change (Representative Agricultural Pathways; Valdivia et al., 2015) linked to global change scenarios (Shared Socio-economic Pathways, SSPs; Kriegler et al., 2012) under which impacts and adaptation studies can be conducted (Vervoort et al., 2014, Rosenzweig et al., 2013b).

### 5.2 Recommendations for simulating adaptation

Current good practice in adaptation studies involves inclusion of autonomous adaptation, since this avoids over-estimation of impacts. Less common, but equally important, is comparison of the effect of any future adaptations to their historical counterparts. As outlined above, adaptation tends to be over-estimated when comparing a non-adapted historical period with an adapted future period. Future directions for modelling adaptation include:

i. New methods are needed in order to permit a broader range of adaptations options to be assessed. Model limitations currently preclude a comprehensive assessment of adaptation, skewing evidence towards a few practices that can be simulated with confidence, rather than covering what is really relevant in specific socio-economic or environmental contexts. Generally, the absence of explicitly representing management as a response to variable conditions (e.g. Hutchings et al. 2012, Waha et al., 2012, van Bussel et al. 2015) in future projections make simulations of adaptation difficult. In addition, Beveridge et al. (submitted) present some promising ways of making crop modelling adaptation studies both more locally relevant and climate-informed.
ii. New methods are also needed to compute adaptation benefits, since crop modelling studies typically do not usually account for technological development (but see Glotter and Elliott, 2016), thereby underestimating the effectiveness of adaptation.
iii. Improved simulation of adaptation through better representation of processes. Ongoing crop model improvement is important (Section 3.2). Many areas need attention, for example sensitivity of climate impacts to nitrogen treatments and inclusion of the response of nitrogen dynamics to elevated CO_2_ (Vanuytrecht and Thorburn, 2017). More generally, research needs to address the lack of consensus on the nature and magnitude of essential processes to be captured in crop models and assess the variation in essential processes with environmental conditions (Fronzek et al., this issue, provide a good example).

## 6 Towards targeted use of models

Ongoing work to improve crop models their use in ensembles is clearly important. However, we argue that innovative approaches to impact and risk assessments will also be needed to address the challenges faced by crop-climate modelling. The Paris Agreement has brought into sharp focus the need to address adaptation and mitigation jointly (see Section 2.3). It has reignited scientific interest in sub-two degree global mean temperature targets and prompted a need for risk assessments that can differentiate between 1.5 and 2.0 degrees of global warming. Detecting systematic differences in crop yields at 1.5 vs 2.0 degrees of warming is currently difficult because the range of model results stemming from methodological choices and spatial variability is large (Schleussner et al., 2016; and Fig. 3 below).

The use of multiple perspectives, as explored in Section 2.4, can help to set appropriate systems boundaries and improve the robustness of risk assessments. However, this approach is of little use unless the various perspectives can be addressed satisfactorily within a single framework or methodology in order to robustly address a key question and/or decision. We now present three areas of progress and potential in this kind of targeted use of models.

### 6.1 Working with stakeholders to identify the timing of risks

The current decade has seen an increasing focus in climate science on identifying the timing of changes in climate (Joshi et al., 2011). This contrasts with the more traditional framing that asks “what will happen at a given time in the future?” Given the large uncertainties that exist, the result of these traditional assessments can often lack utility (Challinor et al., 2007). The more recent focus on timing of risks means that uncertainty is expressed using time intervals, rather than ranges of temperature or crop yield. As a result, these new methods can answer the question “for a given important change in climate, or subsequent impact, when are changes likely to be seen?” By comparing the pace of climate change with the pace of autonomous adaptation, these new methods are generating information on the timing of risks to food production systems (Vermeulen et al., 2013, Rippke et al., 2016).

With the shift in methods towards timing, the focus of adaptation studies can now be ‘by when do key adaptations need to be in place?’ This approach helps in moving from analysis to action (Campbell et al., 2016). In some cases the indications are that food systems are not keeping pace with climate change, as is the case for maize breeding systems in Africa, where the warming that occurs between breeding and final seed usage will result in an unintentionally shorter crop duration (Challinor et al., 2016). Others indicate that more long-term transformations of agricultural systems are needed as land becomes unsuitable for current crops (Rippke et al., 2016). These are exactly the kind of issues that risk assessments need to address. Quantify the timing of interconnected risks (Section 2.2) is likely to be particularly challenging.

With the focus on timing of adaptation comes increasing stakeholder relevance. Furthermore, stakeholders are often needed for robust research results, particularly where understanding of decision-making processes and priorities is required (Lorenz et al., 2015). The MACSUR project has identified agreements on goals with a wide range of stakeholders as a main challenge for European risk assessment (Köchy et al., 2017).

Participatory stakeholder approaches to modelling have taken a variety of innovative forms (Whitfield and Reed, 2012). Vandewindekens et al. (this issue) describe a method of stakeholder input informing a semi-quantitative modelling approach. These participatory approaches have been shown to bring about benefits of improved contextual calibration and decision-making relevance as well as subsequent trust in, and action on, the emergent evidence bases produced by the research (Chaudhury et al., 2013, Reed, 2008, Prell et al., 2013). In summary, engagement with stakeholders is critical if the research is to have a practical risk management or adaptation outcome.

### 6.2 Thinking outside the gridbox

Long-standing approaches to crop-climate modelling ask “what is the change in yield due to climate change in this location and how might cropping systems adapt?” We argue here that it is important to ask different and more useful questions of our modelling studies, using a wide range of methods and information sources. This includes recognising the potential value of interpreting climate model data both with and without using a crop model.

Downscaling is often cited as a method for making crop-climate model output more relevant to stakeholders. However, climate model outputs are not primarily maps, since they do not contain geographic features in the way in which we are accustomed to reading them. Rather, they are information with applicability at spatial scales that depend upon the climate itself, which are usually greater than the domain of that grid cell (Hewitson and Crane, 1996). Crop modelling studies either use the grid on which the input climate simulations were generated, or they downscale those data to a more relevant spatial scale. A range of downscaling methods exist, each with its pros and cons (Wilby and Wigley, 1997). Downscaling is often combined with bias correction, whereby the output of climate models is corrected towards observations (see Section 4.1.1).

Use of native (i.e. non-downscaled) or downscaled climate model grids is a reasonable way of determining impacts and conducting risk analysis. However, it may not be the best way in some situations. As climate models increase their resolution we might expect increases in skill (Challinor et al., 2009), but even this is not a simple or guaranteed process (Garcia-Carreras et al., 2015). Additionally, impact models have their own spatial scale issues that make comprehensive global assessments difficult, and regional-scale information important (Challinor et al., 2014a). Whilst downscaling techniques are regularly applied when field-scale models are used (Vanuytrecht et al., 2016, Vanuytrecht et al., 2014), they nonetheless potentially add bias and are a source of uncertainty.

“Thinking outside the gridbox” is a broad term that tries to capture the need to critically assess the use of climate model output and avoid the presumption that analyses should begin and end with gridded output. This is not a matter of further processing or aggregating gridbox data, but rather of recognising the inherent limitations of it and extracting the maximum information content from the data. Approaches used include non-spatial representations of impacts, as is common in many studies (e.g. quantification of incidence of crop failure rates, Parkes et al., 2015); analysis of collected gridcell data (e.g. Challinor et al., 2010), as opposed to being overly explicit geographically; and use of crop-climate indices (Trnka et al., 2011). In particular the term conveys targeted analyses that employ a range of linked methods and have relatively broad systems boundaries. Challinor et al. (2016) present an example of this approach, by using i. data on the breeding and dissemination of new crop varieties; ii. crop-climate indices, with uncertainty analysis to identify the time at which a climate change signal emerges from current observed variability (see Section 6.1); and iii. ‘traditional’ crop modelling. These methods were used to target crop breeding applications by calculating the spatial and temporal scale of robust crop-climate signals (see Section 6.1 for a brief description of the results of that study).

### 6.3 Increasing transparency and inter-comparability in risk assessments

The various choices (calibrating, running and evaluating models; designing ensembles) faced by a crop modeller when contributing to a risk assessment always result in some limitations. Different choices have different limitations. The purpose of a framework is not only to minimise the limitations, but also to highlight the limitations (see Section 2.4). However, frameworks are often implicit and justification of modelling choices is often missing from crop-climate studies (White et al., 2011), which makes it difficult to compare different studies directly. The identification of consensus views can be supported by clear critical evaluation of methodologies and model projections.

Ruiz-Ramos et al. (this issue) use an *ex post* plausibility check in ensemble wheat modelling, which usefully goes some way towards increasing robustness. However, comparability across risk assessment is only possible when some common methods or protocols are used (see e.g. Liu et al., 2016). Systematic assessments of the response of models to carbon dioxide, temperature, water and nitrogen have been suggested as a way to clearly understand and document model performance (Ruane et al., 2014, Rosenzweig et al., 2013b). The response of the model to changes in key input variables should match what is seen in observations, and a systematic comparison method would aid this assessment.

Inter-comparability of studies needs to go beyond the choice and performance of models. Frameworks and assumptions need to be clearly stated. For example, whilst crop-climate ensembles quantify uncertainty in weather data, it is less common to consider uncertainty in soil data – yet it may be a major driver of variation in yield (Folberth et al., 2016) MIPs are well-placed to take a leading role in the development of standardised approaches to presenting limitations and assumptions (Müller et al., 2017, Hoffmann et al., 2016, Rosenzweig et al., 2013a). A number of option exist: a common uncertainty reporting format can be used to make explicit the assumptions upon which the results of a study are contingent (Wesselink et al., 2014, see especially Supplementary Material ESM3). Alternatively, studies might be assessed against a set of criteria, such as those in Section 1 of the supplementary information.

## Appendix A Supplementary Information

Supplementary material Image 1

## Footnotes

1 https://www.theccc.org.uk/uk-climate-change-risk-assessment-2017/.
