# Supplementary material for: Improving the use of crop models for risk assessment and climate change adaptation
Source: Agric Syst. 2018 Jan;159:296–306. doi: 10.1016/j.agsy.2017.07.010 (PMC5738966; doi:10.1016/j.agsy.2017.07.010)
Supplement: Supplementary file 1 — Supplementary material [file mmc1.docx]

**Towards improved risk assessment and climate change adaptation with crop models**

**Supplementary information**

Challinor et al. (2017)

**Contents**

Section Page

1. Criteria for application of crop models to impacts, adaptation and risk assessment 1

2. Good practice in model and ensemble choices 4

3. Good practice in calibrating and running crop models 6

4. Good practice in model evaluation and interpretation 10

**1. Criteria for application of crop models to impacts, adaptation and risk assessment**

Problems encountered when conducting risk assessments with crop models broadly into three inter-related categories: failing to target a societally-relevant question in an actionable way; inadequate framing or methodology for such a question; and specific model mis-use. Each of the following summary criteria addresses one or more of the issues. The lists are somewhat aspirational. Not all studies will do each of the items equally well, and likely none do them all. Sometimes the issues can be addressed quantitatively, other times only qualitatively. For some items the list will serve as a way of identifying the limitations of the study.

**Criteria for all crop modelling studies**

1. **The crop model used, and the processes simulated, should be of appropriate complexity** given the evidence from available data and the spatial scale of the simulations (see section 2.1 of this document). This helps to avoid overtuning during the calibration process, especially if a broad range of observed data is used. Different models were developed to address different questions. High complexity is warranted where yield-determining processes are demonstrably complex. Field scale models are often used at spatial scales greater than those at which they were developed for, implying potential aggregation error.
2. **Ensembles should be formed from a well-justified set of models and input data** (see section 2.2 of this document). Whist all ensembles are to some extent ensembles of opportunity, they should not be ad hoc. Rather, they should be selected according to skill; and/or provide a reasonable estimate of the underlying distribution of probabilities, or of the expected mean response to climate. It is important to assess the full range of uncertainties (e.g. to understand contributions from weather vs soils; or use of a number of methods to remove a yield technology trend. Wallach et al. (2016a) present a valuable set of recommendations for ensemble modelling.
3. **Bias correction of climate model data should always be carried out**, unless the bias is provably small (see section 2.3 of this document). The assumptions underpinning the method of choice should always be reported, and whenever possible more than one method should be used.
4. **Projections should include uncertainty estimates. The method for quantifying uncertainty should permit assessment of the realism of the resulting ranges.** This assessment is necessarily subjective, and efforts should be made to make clear a range of expert views.
5. **The model used should be evaluated using historical observed data** (see section 4 of this document)**.** A broad range of data (not just yields) over a broad range of environments should be sought and used in evaluating crop models, and error checking of the data is important.
6. **Model projections and methodologies should be critically evaluated and the limitations of the study made explicit.** A model can never be considered entirely valid; evaluation is an ongoing process. Ultimately it is interconnected learning from models, data and theory that result in sound science. Ruiz-Ramos et al. (this issue) provide an example of critical plausibility assessment in ensemble modelling. Engagement with stakeholders can be an important part of the process of critical evaluation.
7. **The assumptions underlying the results of the study should be explicit.** A common uncertainty reporting format can be used to achieve this.
8. **Assessments of climate change impacts should include autonomous adaptation**; otherwise impacts will likely be over-estimated.
9. **The simulations carried out should be documented** in sufficient detail to demonstrate the extent to which the above criteria have been met, and to ensure reproducibility of the work carried out. For example, Hoffman et al. (this issue) use web references to ensure that model documentation is available.

**Additional criteria for adaptation studies and risk assessments**

1. **Assessments of risk need broad system boundaries**. Climate change risks are fundamentally interconnected with each other and are transmitted across borders and across sectors. They also interact in complex ways with each other and with non-climatic risks. If these relationships are not assessed explicitly within a study then existing literature should be used to comment on their implications. Guzman et al. (this issue) and Elliot et al. (this issue) provide exemplar studies interactions and assessment of economic impacts, respectively.
2. **Engagement with stakeholders is critical** if the research aims to have a practical risk management or adaptation outcome. Projections should include uncertainty estimates that aid end-users to evaluate the utility and reliability of the results. Information on the timing of risks is one area that is often lacking and where recent progress in methods is beginning to yield results. Example of good practice: Vanwindekens et al. (this issue).
3. **The effect of any future adaptations should be compared directly to their historical counterparts**, since adaptation tends to be over-estimated by the common practice of comparing a non-adapted historical period with an adapted future period.
4. **A critical assessment of all the adaptations modelled should be presented**, since all methods have inherent limitations.
5. **Adaptation should not be assessed without any reference to mitigation.** The challenges of adapting agricultural systems to climate change are not independent of the need to reduce emissions. If possible, this should at least be recognised by calculation or literature-based estimation of the emissions associated with the adaptation options explored in a study. Example of good practice: Tian et al. (this issue).

**2. Good practice in model and ensemble choices**

**2.1** **Model choice: appropriate complexity and spatial scale**

Crop models vary widely in the processes they include and the complexity of how these are simulated. Choices made by model developers about how crop physiology is represented will lead to differences amongst models. In recent years it has become common to use crop models more widely including in climates outside the range the models have been designed and calibrated for, e.g. for model intercomparison projects or climate change studies. If a model is to obtain the right answer for the right reason, thus engendering confidence when it is run out-of-sample, then it is essential to perform well historically whilst avoiding overtuning. If a model is overly simple then it may not capture the correct responses to a complex environment. If it is overly complex then it will be difficult to adequately constrain the full parameter set.

There are two broad approaches to judging model complexity. The process perspective is predicated on expert judgement stating that a particular process is important in the environment being simulated. This approach makes excellent use of fundamental science. However, excess complexity can result from inclusion of many processes with perceived *a priori* importance. To address this, objective criteria for model component selection can be developed (Adam et al., 2012 provide a protocol for this). The second approach is a data-driven perspective that asks: what is the evidence from the data that this process is important? In practice both of these approaches are needed, since knowledge of G x E x M (interactions between genotype, environment, and management) is incomplete, and datasets will never be ideal (see e.g.Challinor et al., 2014a).

**2.2** **Ensembles need to be formed from well-justified models and input data**

However, not all simulations are equally likely, nor even necessarily realistic. Model ensembles using crop and/or climate models are usually “ensembles of opportunity” – i.e. all candidate simulations are accepted. This presents a challenge: there is no evidence that the ensemble members are a random sample of the underlying (“true”) distribution. In other words, we cannot assume that the full range of possible events (area *a+b+c+d* in Fig. 3 of the main paper) is captured, nor do we know that unrealistic simulations are absent (area *e* in Fig. 3). Methods for tailoring uncertainty estimates to the particular prediction situation of a given study have been developed (Wallach et al., 2016b), and are likely to prove useful in improving risk assessment.

Multi-model ensembles can help to measure the component of uncertainty in crop model projections that cannot be quantified by single-model applications. The choice of methods has a significant impact on the results: where multiple crop models are used (Müller et al., 2015, Asseng et al., 2013), or where parameter perturbations are used in single models (Challinor et al., 2005, Ann-Kristin et al., 2013), crop model uncertainty can be of similar magnitude to climate uncertainty. However, where crop parameter values are more constrained within a single model (Challinor et al., 2009b), or where initial conditions in climate simulations are important (Vermeulen et al., 2013), reported uncertainty in crop simulation can be relatively small.

Ensembles of opportunity, whether single- or multi-model, have been the norm in crop-climate modelling, perhaps both because it is efficient use of time and because objective methods of model selection have only recently begun to be developed. Model selection could improve upon this norm, if based on appropriate criteria and if models were freely accessible and well documented. Accurate quantification of uncertainty involves both assessment of skill of individual ensembles members, and assessment of the extent to which the full range is deemed realistic.

**2.3 Bias correction**

Whatever method of justification is used for the choice of climate model, some climate model error will always be present. Bias correction is therefore needed when errors or incorrectly simulated features in global or regional climate models affect the correct simulation of crop (e.g. primary productivity, yield, leaf area) or farmer (e.g. planting date, replanting) behaviour (Garcia-Carreras et al., 2015, Berg et al., 2010, Guan et al., 2015). A variety of bias correction methods exist (Hawkins et al., 2013b, Ines and Hansen, 2006, Themeßl et al., 2011). By definition, however, no bias correction method is capable of fully correcting errors in GCMs while at the same time keeping the climate model physics intact (see Ehret et al., 2012 for a complete discussion). Post-processing of climate model data often also includes downscaling.

Recent crop modelling studies demonstrate that the uncertainty in the choice of bias correction method when projecting climate change impacts can be substantial (Hawkins et al., 2013a, Ramirez-Villegas and Challinor, 2016) and similar findings have been reported in the hydrological modelling community (Themeßl et al., 2011, Themeßl et al., 2012, Quintana Seguí et al., 2010). Therefore, while there is agreement in the crop modelling community that bias correction of climate model output is needed, crop-climate modelling studies need to (i) understand and report the assumptions going into the method of choice, and (ii) whenever possible use more than one method. The development of automated bias correction portals such as CCAFS-Climate (Navarro-Racines et al., 2016, http://ccafs-climate.org/data_bias_correction/, 2016) and of global bias-corrected datasets (e.g. the ISI -MIP dataset, Hempel et al., 2013) are crucial steps toward facilitating the sampling of this critical source of uncertainty.

**3 Good practice in calibrating and running crop models**

**3.1 Selection of input data**

The selection of input data, i.e. weather, soil etc. requires consideration of a number of factors. There is often a trade-off between data quality and spatial coverage. Site-based weather station data is typically better quality than gridded weather data (Van Wart et al., 2013). However, in order for site-based studies to provide information at the regional scale the results must be scaled up, which creates additional uncertainty (Hansen and Jones, 2000, Ewert et al., 2011). In addition, weather station data can be of low quality, contain missing values or fail to provide all of the required variables, again causing uncertainty (Rivington et al., 2006). The most appropriate combination of data therefore depends on the region of interest and the aims of the study (Grassini et al., 2015).

It is important to account for the uncertainty in the model input data. Crop modelling studies often consider uncertainty in weather data but it rare for uncertainty in management practices to be taken into account (White et al., 2011). Where error variances are known the full range of data can be used within a statistical framework that downweights poor quality data. Where errors are difficult to quantify, but are known to some extent qualitatively, it can be better to use a small sample of different data sets most suited to the study region than to use a large sample of data sets without careful consideration of their quality and suitability. It is also possible to make assumptions about standard errors in observed data (e.g. Alderman et al., 2015). Taking soil data as an example, the data sets selected should ideally have a high density of observations in the study region and should only use pedo-transfer functions that have been developed and tested for the range of soil textures within the study region (Hodnett and Tomasella, 2002).

In some cases, a high quality data set covering the study region is not readily available. Where this is the case, every effort should be made to obtain realistic data. A typical example is management information such as planting dates in regional or global studies (Angulo et al., 2013). More realistic data can be obtained by consulting local experts, making estimates based on knowledge of crop requirements or by supplementing available data using information from published or grey literature (Waha et al., 2012). In addition, the impact of using lower quality input data on the study outcomes should be evaluated, either by performing a sensitivity study or by comparing results at sites where higher quality data is available (Challinor et al., 2014a).

**3.2 Calibration**

The process of obtaining appropriate model parameter sets is called calibration. The value of each parameter is specified either by direct measurement or in a calibration procedure where parameter values are adjusted to improve the match between model outputs and observations. The method used for calibration has been shown to affect both the parameter values obtained and the simulated impact of climate change (Guillaume et al., 2011, Angulo et al., 2013). The main danger when calibrating a crop model is overtuning, i.e. having parameter values that give skilful results, but are not suitable for the full range of environments and varieties to be simulated, or which do not represent the processes occurring within the crop (Challinor et al., 2009a).

The key to avoiding overtuning is data. The data used for calibration must represent behaviour across the range of environments and varieties to be simulated (Therond et al., 2011, Klein et al., 2012), and cover a sufficiently large number of variables and crop growth dynamics (Guérif and Duke, 2000, Guillaume et al., 2011). It is also important to have both model equations and parameter values that are well constrained by the data (Wallach, 2011, Craufurd et al., 2013). If a crop model is mis-specified (e.g. incorrect equations or parameter values), a calibration procedure will not necessarily result in an ability to reproduce observations across the test dataset or in a different dataset (Guillaume et al., 2011, Wallach, 2011). Ideally calibration procedures should use observations of multiple crop characteristics rather than just crop yield (Guérif and Duke, 2000, Guillaume et al., 2011). They should also incorporate internal consistency checks based on observations (e.g. Challinor et al., 2004).

The data available will always have its limitations in terms of quantity and quality. It is also likely that multiple parameter sets are supported by the data (the equifinality thesis, e.g. Beven, 2006). Therefore, an ensemble of parameter sets should be used, and sensitivity analysis conducted, in order to take into account this uncertainty (e.g. Challinor and Wheeler, 2008, Klein et al., 2012). In addition, the uncertainty in the observations themselves should be considered as it can have a large impact on the selected parameter values and therefore the simulated yields (e.g. Confalonieri et al., 2016).

Finally, care must be taken when calibrating crop models for use outside the current range of environments and varieties (e.g. in order to simulate crop response to climate change or varietal adaptation). Estimating how parameter values might change as new varieties are developed is especially difficult. It typically relies upon observations of the current range of variability coupled with expert knowledge (Ramirez-Villegas et al., 2015).

**3.3 Documentation of models and simulations**

Documentation of models and of their evaluation is often lacking or based on amendment to the original model documentation, and evaluation of the simulated responses is often limited (Rotter et al., 2011), especially for responses to elevated CO_2_ (Vanuytrecht & Thorburn, 2017). The temperature routines of most wheat models have been recently summarised (Alderman et al., 2013), providing both a useful intercomparisson and a multiple documentation for these processes. However, in general documentation of crop models is variable. For example, the most recent comprehensive document describing all key processes in the CERES wheat and maize models of the DSSAT suite dates back to Jones et al. (1983), whereas the IXIM-maize model’s documentation (also in the DSSAT suite) dates only a few years back (Lizaso et al., 2011). Poor documentation limits the ability to reach consensus on captured responses among models, reduces the reproducibility of the study in question, and limits our ability to decide how suitable a given model is for a certain study. Lack of documentation also causes models to become ‘black boxes’ and hence limits the contribution of a study to knowledge and society. As an example, documentation of captured CO_2_ responses is often out-dated or lacks sufficient detail (see (White et al., 2011). Furthermore, as models are continually developed, different versions are often used by different modellers. Without documentation, it would be easy to make incorrect inferences regarding model performance.

Model documentation needs to go beyond expressing and justifying model equations and parameter choices, to cover evaluation of models and modelling components for all modelled processes. Shared standards are a clear priority in crop modelling (White et al., 2011).

**4 Good practice in model evaluation and interpretation**

Models need to be tested on historical field experiments in order to determine future trustworthiness. Whilst this is often referred to in the literature as “validation”, we contend that there is no procedure that can validate a model for future use; there is only ongoing evaluation (we use inverted commas for this reason). Comparing models to data only provides information on model performance under those specific conditions. Demonstrating suitability should include a thorough, well-structured evaluation process, as advocated by e.g. White et al. (2011). In addition to increasing confidence in simulations, model evaluation can identify processes in the model that need improvement.

The fundamentals of model evaluation have long been understood (see section 2.3 of Sinclair and Seligman, 2000) and are strongly related to the practice of calibrating and running crop models. They include for example, separation of calibration and evaluation data sets or testing out of sample. However, even this well-understood practice is not always carried out: the White et al. (2011) analysis of crop modelling studies found that “where evaluations were presented, these often relied on comparisons of means and variances for historic yields, rather than using cross validation”.

Recently, systematic assessments of the response of models to carbon dioxide, temperature, water and nitrogen have been suggested as a way to clearly understand and document model performance (Ruane et al., 2014). Clearly, the response of the model to changes in key input variables should match what is seen in observations, and a systematic comparison method would aid this assessment. Given the importance of extreme events under climate change, and the key role of stability of supply in food security, interannual variability in yields is also very important; yet it is often left unevaluated (Challinor et al., 2014b).

It is not only end-of-season values, such as those discussed above, that are important: there is a need to evaluate multiple model outputs at various stages in the growing season in order to test whether the model is faithfully representing the underlying processes (Sinclair and Seligman, 2000, Wesselink et al., 2014, Challinor et al., 2014a). Ultimately it is interconnected learning from models, data and theory that result in sound science.

The data used for model evaluation can also be a source of model prediction error. The data should therefore be error checked and the uncertainty in the data should be taken into account in interpreting results. When using regional observed yields the simulated yields should where possible be compared to each available data set. Both national and sub-national data should be used where possible, since national data may mask important spatial variations whilst being of higher quality than sub-national data (Watson et al., 2014). Different data sets at the same spatial scale should also be used if possible, as there can be significant disagreement between them (Grassini et al., 2015). In addition, several methods of aggregation (Porwollik et al. in press) and of removing the technology trend from the yield time series (e.g. Elliott et al., 2015), if the technology trend cannot be modelled itself as e.g. by Glotter and Elliott (2016) should be used to check that the method chosen does not affect the results of the study.

The multitude of assumptions, parameters, input data, data processing, metrics and reference data make a comparison of model evaluations difficult. A standardized evaluation routine, including freely accessible modelling protocols and input data (Elliott et al. 2015), open source evaluation processing pipeline and a web interface that allows any modeller to evaluate their model’s performance against reference data and other tested models is thus desirable. The work of Müller et al. (2017) provides an example for such a standardized evaluation procedure and online tool (https://mygeohub.org/resources/ggcmevaluation) for global gridded crop models.

**References**

ADAM, M., BELHOUCHETTE, H., CORBEELS, M., EWERT, F., PERRIN, A., CASELLAS, E., CELETTE, F. & WERY, J. 2012. Protocol to support model selection and evaluation in a modular crop modelling framework: An application for simulating crop response to nitrogen supply. *Computers and Electronics in Agriculture,* 86**,** 43-54.

ALDERMAN, P., QUILLIGAN, E., ASSENG, S., EWERT, F. & REYNOLDS, M. Proceedings of the Workshop Modeling Wheat Response to High Temperature. El Batán, Mexico, 2013. Mexico: International Maize and Wheat Improvement Center (CIMMYT).

ANGULO, C., RÖTTER, R., LOCK, R., ENDERS, A., FRONZEK, S. & EWERT, F. 2013. Implication of crop model calibration strategies for assessing regional impacts of climate change in Europe. *Agricultural and Forest Meteorology,* 170**,** 32-46.

ANN-KRISTIN, K., ANDREW, J. C., ED, H. & SENTHOLD, A. 2013. Influences of increasing temperature on Indian wheat: quantifying limits to predictability. *Environmental Research Letters,* 8**,** 034016.

ASSENG, S., EWERT, F., ROSENZWEIG, C., JONES, J. W., HATFIELD, J. L., RUANE, A. C., BOOTE, K. J., THORBURN, P. J., ROTTER, R. P., CAMMARANO, D., BRISSON, N., BASSO, B., MARTRE, P., AGGARWAL, P. K., ANGULO, C., BERTUZZI, P., BIERNATH, C., CHALLINOR, A. J., DOLTRA, J., GAYLER, S., GOLDBERG, R., GRANT, R., HENG, L., HOOKER, J., HUNT, L. A., INGWERSEN, J., IZAURRALDE, R. C., KERSEBAUM, K. C., MULLER, C., NARESH KUMAR, S., NENDEL, C., O/'LEARY, G., OLESEN, J. E., OSBORNE, T. M., PALOSUO, T., PRIESACK, E., RIPOCHE, D., SEMENOV, M. A., SHCHERBAK, I., STEDUTO, P., STOCKLE, C., STRATONOVITCH, P., STRECK, T., SUPIT, I., TAO, F., TRAVASSO, M., WAHA, K., WALLACH, D., WHITE, J. W., WILLIAMS, J. R. & WOLF, J. 2013. Uncertainty in simulating wheat yields under climate change. *Nature Clim. Change,* 3**,** 827-832.

BERG, A., SULTAN, B. & DE NOBLET-DUCOUDRÉ, N. 2010. What are the dominant features of rainfall leading to realistic large-scale crop yield simulations in West Africa? *Geophys. Res. Lett.,* 37**,** L05405.

BEVEN, K. 2006. A manifesto for the equifinality thesis. *Journal of Hydrology,* 320**,** 18-36.

CHALLINOR, A., MARTRE, P., ASSENG, S., THORNTON, P. & EWERT, F. 2014a. Making the most of climate impacts ensembles. *Nature Clim. Change,* 4**,** 77-80.

CHALLINOR, A. J., EWERT, F., ARNOLD, S., SIMELTON, E. & FRASER, E. 2009a. Crops and climate change: progress, trends, and challenges in simulating impacts and informing adaptation. *Journal of Experimental Botany,* 60**,** 2775-2789.

CHALLINOR, A. J., WATSON, J., LOBELL, D. B., HOWDEN, S. M., SMITH, D. R. & CHHETRI, N. 2014b. A meta-analysis of crop yield under climate change and adaptation. *Nature Clim. Change,* 4**,** 287-291.

CHALLINOR, A. J. & WHEELER, T. R. 2008. Use of a crop model ensemble to quantify CO2 stimulation of water-stressed and well-watered crops. *Agricultural and Forest Meteorology,* 148**,** 1062-1077.

CHALLINOR, A. J., WHEELER, T. R., CRAUFURD, P. Q., SLINGO, J. M. & GRIMES, D. I. F. 2004. Design and optimisation of a large-area process-based model for annual crops. *Agricultural and Forest Meteorology,* 124**,** 99-120.

CHALLINOR, A. J., WHEELER, T. R., HEMMING, D. & UPADHYAYA, H. D. 2009b. Ensemble yield simulations: crop and climate uncertainties, sensitivity to temperature and genotypic adaptation to climate change. *Climate Research,* 38**,** 117-127.

CHALLINOR, A. J., WHEELER, T. R., SLINGO, J. M. & HEMMING, D. 2005. Quantification of physical and biological uncertainty in the simulation of the yield of a tropical crop using present-day and doubled CO2 climates. *Philosophical Transactions of the Royal Society B: Biological Sciences,* 360**,** 2085-2094.

CONFALONIERI, R., BREGAGLIO, S. & ACUTIS, M. 2016. Quantifying uncertainty in crop model predictions due to the uncertainty in the observations used for calibration. *Ecological Modelling,* 328**,** 72-77.

CRAUFURD, P. Q., VADEZ, V., JAGADISH, S. V. K., VARA PRASAD, P. V. & ZAMAN-ALLAH, M. 2013. Crop science experiments designed to inform crop modeling. *Agricultural and Forest Meteorology,* 170**,** 8-18.

EHRET, U., ZEHE, E., WULFMEYER, V., WARRACH-SAGI, K. & LIEBERT, J. 2012. Should we apply bias correction to global and regional climate model data? *Hydrology and Earth System Sciences Discussions,* 9**,** 5355-5387.

ELLIOTT, J., MÜLLER, C., DERYNG, D., CHRYSSANTHACOPOULOS, J., BOOTE, K. J., BÜCHNER, M., FOSTER, I., GLOTTER, M., HEINKE, J., IIZUMI, T., IZAURRALDE, R. C., MUELLER, N. D., RAY, D. K., ROSENZWEIG, C., RUANE, A. C. & SHEFFIELD, J. 2015. The Global Gridded Crop Model Intercomparison: data and modeling protocols for Phase 1 (v1.0). *Geosci. Model Dev.,* 8**,** 261-277.

EWERT, F., VAN ITTERSUM, M. K., HECKELEI, T., THEROND, O., BEZLEPKINA, I. & ANDERSEN, E. 2011. Scale changes and model linking methods for integrated assessment of agri-environmental systems. *Agriculture, Ecosystems & Environment,* 142**,** 6-17.

GARCIA-CARRERAS, L., CHALLINOR, A. J., PARKES, B. J., BIRCH, C. E., NICKLIN, K. J. & PARKER, D. J. 2015. The Impact of Parameterized Convection on the Simulation of Crop Processes. *Journal of Applied Meteorology and Climatology,* 54**,** 1283-1296.

GRASSINI, P., VAN BUSSEL, L. G. J., VAN WART, J., WOLF, J., CLAESSENS, L., YANG, H., BOOGAARD, H., DE GROOT, H., VAN ITTERSUM, M. K. & CASSMAN, K. G. 2015. How good is good enough? Data requirements for reliable crop yield simulations and yield-gap analysis. *Field Crops Research,* 177**,** 49-63.

GUAN, K., SULTAN, B., BIASUTTI, M., BARON, C. & LOBELL, D. B. 2015. What aspects of future rainfall changes matter for crop yields in West Africa? *Geophysical Research Letters,* 42**,** 8001-8010.

GUÉRIF, M. & DUKE, C. L. 2000. Adjustment procedures of a crop model to the site specific characteristics of soil and crop using remote sensing data assimilation. *Agriculture, Ecosystems &amp; Environment,* 81**,** 57-69.

GUILLAUME, S., BERGEZ, J.-E., WALLACH, D. & JUSTES, E. 2011. Methodological comparison of calibration procedures for durum wheat parameters in the STICS model. *European Journal of Agronomy,* 35**,** 115-126.

HANSEN, J. W. & JONES, J. W. 2000. Scaling-up crop models for climate variability applications. *Agricultural Systems,* 65**,** 43-72.

HAWKINS, E., FRICKER, T. E., CHALLINOR, A. J., FERRO, C. A. T., KIT HO, C. & OSBORNE, T. M. 2013a. Increasing influence of heat stress on French maize yields from the 1960s to the 2030s. *Global Change Biology***,** n/a-n/a.

HAWKINS, E., OSBORNE, T. M., HO, C. K. & CHALLINOR, A. J. 2013b. Calibration and bias correction of climate projections for crop modelling: an idealised case study over Europe. *Agricultural and Forest Meteorology,* 170**,** 19-31.

HEMPEL, S., FRIELER, K., WARSZAWSKI, L., SCHEWE, J. & PIONTEK, F. 2013. A trend-preserving bias correction &ndash; the ISI-MIP approach. *Earth Syst. Dynam.,* 4**,** 219-236.

HODNETT, M. G. & TOMASELLA, J. 2002. Marked differences between van Genuchten soil water-retention parameters for temperate and tropical soils: a new water-retention pedo-transfer functions developed for tropical soils. *Geoderma,* 108**,** 155-180.

<HTTP://CCAFS-CLIMATE.ORG/DATA_BIAS_CORRECTION/>. 2016. *Bias Correction Data* [Online].

INES, A. V. M. & HANSEN, J. W. 2006. Bias correction of daily GCM rainfall for crop simulation studies. *Agricultural and Forest Meteorology,* 138**,** 44-53.

JONES, C. A., RITCHIE, J. T., KINIRY, J. R., GODWIN, D. C. & OTTER, S. I. 1983. The CERES Wheat and Maize Models. Proceedings of the International Symposium on Minimum Data Sets for Agrotechnology Transfer. Patancheru, India.

KLEIN, T., CALANCA, P., HOLZKÄMPER, A., LEHMANN, N., ROESCH, A. & FUHRER, J. 2012. Using farm accountancy data to calibrate a crop model for climate impact studies. *Agricultural Systems,* 111**,** 23-33.

LIZASO, J. I., BOOTE, K. J., JONES, J. W., PORTER, C. H., ECHARTE, L., WESTGATE, M. E. & SONOHAT, G. 2011. CSM-IXIM: A New Maize Simulation Model for DSSAT Version 4.5 All rights reserved. No part of this periodical may be reproduced or transmitted in any form or by any means, electronic or mechanical, including photocopying, recording, or any information storage and retrieval system, without permission in writing from the publisher. *Agronomy Journal,* 103**,** 766-779.

MÜLLER, C., ELLIOTT, J., CHRYSSANTHACOPOULOS, J., DERYNG, D., FOLBERTH, C., PUGH, T. A. M. & SCHMID, E. 2015. Implications of climate mitigation for future agricultural production,. *Environmental Research Letters,* 10.

NAVARRO-RACINES, C., TARAPUES-MONTENEGRO, J. & RAMIREZ-VILLEGAS, J. 2016. *Bias-correction in the CCAFS-Climate portal: a description of methodologies. ,* Cali, Columbia.

QUINTANA SEGUÍ, P., RIBES, A., MARTIN, E., HABETS, F. & BOÉ, J. 2010. Comparison of three downscaling methods in simulating the impact of climate change on the hydrology of Mediterranean basins. *Journal of Hydrology,* 383**,** 111-124.

RAMIREZ-VILLEGAS, J. & CHALLINOR, A. J. 2016. Towards a genotypic adaptation strategy for Indian groundnut cultivation using an ensemble of crop simulations. *Climatic Change,* 138**,** 223-238.

RAMIREZ-VILLEGAS, J., WATSON, J. & CHALLINOR, A. J. 2015. Identifying traits for genotypic adaptation using crop models. *Journal of Experimental Botany,* 66**,** 3451-3462.

RIVINGTON, M., MATTHEWS, K. B., BELLOCCHI, G. & BUCHAN, K. 2006. Evaluating uncertainty introduced to process-based simulation model estimates by alternative sources of meteorological data. *Agricultural Systems,* 88**,** 451-471.

ROTTER, R. P., CARTER, T. R., OLESEN, J. E. & PORTER, J. R. 2011. Crop-climate models need an overhaul. *Nature Clim. Change,* 1**,** 175-177.

RUANE, A. C., MCDERMID, S., ROSENZWEIG, C., BAIGORRIA, G. A., JONES, J. W., ROMERO, C. C. & DEWAYNE CECIL, L. 2014. Carbon–Temperature–Water change analysis for peanut production under climate change: a prototype for the AgMIP Coordinated Climate-Crop Modeling Project (C3MP). *Global Change Biology,* 20**,** 394-407.

SINCLAIR, T. R. & SELIGMAN, N. A. 2000. Criteria for publishing papers on crop modeling. *Field Crops Research,* 68**,** 165-172.

THEMEßL, J. M., GOBIET, A. & LEUPRECHT, A. 2011. Empirical-statistical downscaling and error correction of daily precipitation from regional climate models. *International Journal of Climatology,* 31**,** 1530-1544.

THEMEßL, M., GOBIET, A. & HEINRICH, G. 2012. Empirical-statistical downscaling and error correction of regional climate models and its impact on the climate change signal. *Climatic Change,* 112**,** 449-468.

THEROND, O., HENGSDIJK, H., CASELLAS, E., WALLACH, D., ADAM, M., BELHOUCHETTE, H., OOMEN, R., RUSSELL, G., EWERT, F., BERGEZ, J.-E., JANSSEN, S., WERY, J. & VAN ITTERSUM, M. K. 2011. Using a cropping system model at regional scale: Low-data approaches for crop management information and model calibration. *Agriculture, Ecosystems & Environment,* 142**,** 85-94.

VAN WART, J., GRASSINI, P. & CASSMAN, K. G. 2013. Impact of derived global weather data on simulated crop yields. *Global Change Biology,* 19**,** 3822-3834.

VERMEULEN, S. J., CHALLINOR, A. J., THORNTON, P. K., CAMPBELL, B. M., ERIYAGAMA, N., VERVOORT, J. M., KINYANGI, J., JARVIS, A., LÄDERACH, P., RAMIREZ-VILLEGAS, J., NICKLIN, K. J., HAWKINS, E. & SMITH, D. R. 2013. Addressing uncertainty in adaptation planning for agriculture. *Proceedings of the National Academy of Sciences,* 110**,** 8357-8362.

WAHA, K., VAN BUSSEL, L. G. J., MÜLLER, C. & BONDEAU, A. 2012. Climate-driven simulation of global crop sowing dates. *Global Ecology and Biogeography,* 21**,** 247-259.

WALLACH, D. 2011. Crop Model Calibration: A Statistical Perspective. *Agronomy Journal,* 103**,** 1144-1151.

WALLACH, D., MEARNS, L. O., RUANE, A. C., RÖTTER, R. P. & ASSENG, S. 2016a. Lessons from climate modeling on the design and use of ensembles for crop modeling. *Climatic Change***,** 1-14.

WALLACH, D., NISSANKA, S. P., KARUNARATNE, A. S., WEERAKOON, W. M. W., THORBURN, P. J., BOOTE, K. J. & JONES, J. W. 2016b. Accounting for both parameter and model structure uncertainty in crop model predictions of phenology: A case study on rice. *European Journal of Agronomy*.

WATSON, J., CHALLINOR, A. J., FERRO, C. A. T. & FRICKER, T. E. 2014. Simulating national-level maize yield in France using a process-based and statistical model: assessing data requirements. *Climatic Change,* Submitted.

WESSELINK, A., CHALLINOR, A., WATSON, J., BEVEN, K., ALLEN, I., HANLON, H., LOPEZ, A., LORENZ, S., OTTO, F., MORSE, A., RYE, C., SAUX-PICARD, S., STAINFORTH, D. & SUCKLING, E. 2014. Equipped to deal with uncertainty in climate and impacts predictions: lessons from internal peer review. *Climatic Change***,** 1-14.

WHITE, J. W., HOOGENBOOM, G., KIMBALL, B. A. & WALL, G. W. 2011. Methodologies for simulating impacts of climate change on crop production. *Field Crops Research,* 124**,** 357-368.

Glotter M, and Elliott J. 2016, Simulating US agriculture in a modern Dust Bowl drought, Nature Plants, 3, 16193, doi: 10.1038/nplants.2016.193.
